# Age as a Dynamic Moderator of Relations between Exposure to Political Conflict and Mental Health in Belfast, Northern Ireland

**DOI:** 10.3390/ijerph19148339

**Published:** 2022-07-08

**Authors:** Christine E. Merrilees, Laura K. Taylor, Marcie C. Goeke-Morey, Peter Shirlow, E. Mark Cummings

**Affiliations:** 1 Psychology Department, State University of New York, Geneseo, NY 14454, USA; 2 School of Psychology, University College Dublin, D04 V1W8 Dublin, Ireland; laura.taylor@ucd.ie; 3 Psychology Department, The Catholic University of America, Washington, DC 20064, USA; goekemorey@cua.edu; 4 School of Histories, Languages, and Cultures, University of Liverpool, Liverpool L69 3BX, UK; p.shirlow@liverpool.ac.uk; 5 Department of Psychology, University of Notre Dame, Notre Dame, IN 46556, USA; edward.m.cummings.10@nd.edu

**Keywords:** developmental psychopathology, TVEM, political violence, dynamic change

## Abstract

Identifying how, when, and under what conditions exposure to political conflict is associated with youth mental health problems is critical to developing programming to help youth exposed to various forms of political violence. The current study uses Time Varying Effects Modeling (TVEM) to examine how relations between exposure to ethno-politically motivated antisocial behavior and mental health problems change as a function of age in a sample of youth from Belfast, Northern Ireland. Young people (*N* = 583, M_age_ 16.51 wave 1, 17.23 wave 2) self-reported their exposure to sectarian antisocial behavior, nonsectarian antisocial behavior, and mental health problems as part of a longitudinal study of youth across multiple neighborhoods in Belfast. The results suggest mental health problems and associations with exposure to sectarian antisocial behavior change in nonlinear patterns throughout adolescence, with the strongest links between exposure to political conflict and mental health between ages 16 and 19. Significant relations between nonsectarian antisocial behavior and mental health problems were not indicated for the full sample but the results suggested a relation emerged in later adolescence for Protestant youth, the historical majority group. The value of this exploratory approach to examining relations between key context and psychological variables for youth in contexts of political tension and violence is discussed.

## 1. Introduction

Understanding the relation between exposure to political violence and youth adjustment is critical to targeting prevention and intervention programs to help youth exposed to the various forms of armed conflict that plague the globe. Utilizing a developmental psychopathology perspective is especially informative for understanding for whom and under what conditions political violence impacts youth adjustment [1,2]. Identifying the nuanced processes associated with time is critical to understanding what should be targeted, as well as when interventions should take place [3,4]. For example, it may be the case that exposure to political violence initially causes a freezing response or a denial of the experience as a form of self-defense. The impact of that exposure may take time to unfold. At the same time, it may be the case that younger youth have a short-term response that differs from older youth who have greater cognitive and meaning making capabilities on average. Some developmental processes may follow simple linear patterns (e.g., increased cognitive ability with age), but the experience of political violence is complex and varies with context-level variables that are also dynamic, potentially altering the meaning and impact of exposure. Thus, using exploratory methods that do not impose specific time constraints on the data may allow unexpected change processes to emerge that lead us to a new understanding of how political violence impacts youth across development.

The current study utilizes Time Varying Effects Modeling (TVEM; [5]) to examine age related changes in the relation between exposure to political violence and mental health for young people in ethnically segregated neighborhoods in post-accord Belfast, Northern Ireland. TVEM allows for the examination of age as a moderator as a continuous function of time. Using a semi-parametric approach, the goal of TVEM is to capture the nature of change in the data that may be more dynamic than the change functions (e.g., linear, quadratic, cubic) typically prescribed in parametric tests, such as multilevel modeling and structural equation modeling. In this way, TVEM is an exploratory approach to examine how the relations between variables change over time and can aid in targeting prevention and intervention programs in terms of developmental age or based on repeated exposure to a stressor, the impacts of which can accumulate over days, months, or years. In other words, it helps to answer questions about when a particular predictor may have a stronger association with an outcome. The current study focuses on links between exposure to political violence and tension and youth mental health problems. Adolescence is a key period, during which internalizing problems, including symptoms of clinical and subclinical depression and anxiety, start to emerge [6]. Even at sub-clinical levels, these mental health symptoms increase the risk for future diagnoses of depression, anxiety, and other mental health and adjustment problems. Thus, identifying key predictors of mental health problems and the timing of when these predictors emerge as significant may be key to targeting intervention and prevention programs.

Within developmental studies on the impacts of political violence and youth, outcomes of interest often focus on internalizing and externalizing problems broadly defined. Although initial studies on the impacts of the political violence on youth suggested behavior problems and school problems were more likely to result from exposure to political violence in Northern Ireland [7], more recent research across conflicts suggests that political violence exposure increases the risk for internalizing problems. For example, a recent meta-analysis of various factors associated with exposure to political violence in young people found a small but significant relation with depression in particular [8]. Multiple studies across conflicts have documented the increased risk for internalizing problems and symptoms [9,10,11].

There have been multiple related concerns about the impact of political violence on mental health problems for youth in Belfast in particular. For example, Tomlison (2007) [12] discussed concern about depressive symptomatology for youth in socially and economically deprived areas of Belfast, in part due to those communities enduring a majority of the violence that occurred during the height of political violence. Concern for the impact of violence exposure on youth also stems from the observation that compared to adults, youth are more likely to be exposed to violence in general [13], as well as to sectarian violence in particular [14]. Research on youth exposure to violence in Northern Ireland also suggests indirect forms of exposure to violence are common [15]. Moreover, recent longitudinal studies on the impact of political tension and violence on youth suggest post-accord experiences of conflict in Belfast do increase the risk for internalizing problems [16,17], but they do not examine if those associations are uniform across adolescence. 

The current study uses data from a longitudinal study on the impacts of exposure to political violence on youth in Belfast, Northern Ireland. The contemporary conditions in Belfast were most recently defined by the Troubles, a 30-year period between the late 1960s and 1990s, during which two main ethnopolitical groups were in conflict over the constitutional state of Northern Ireland [18]. Within Northern Ireland, there are two main ethnopolitical groups, colloquially referred to as having a Catholic or Protestant ‘community background’. Protestants represent the historic majority group and Catholics the minority group. An example of this dynamic was the Protestant-dominated government’s prevention of Catholics having equal access to certain things, such as housing and education, compared to Protestants [19]. The Troubles resulted in armed conflict between state forces, paramilitary groups, and civilians, resulting in 3600 deaths, 20,000–30,000 imprisoned, and roughly 50,000 injured [18,20,21]. In 1998, a comprehensive peace agreement, known as the Belfast/Good Friday Agreement, was signed, but segregation, tensions between communities, and recurrent flares of violence persist in many parts of Belfast. 

The goals of the current study are to explore if and how the relationship between exposure to political tension and mental health problems changes through adolescence. To do this, we utilize TVEM to allow changes in that relationship to emerge from the data. In this way, there is no pre-planned notion about the shape of change imposed on the data. Age-based changes in relations between community violence that is not politically motivated is also examined to explore differences in these types of community tension. Given the history of majority and minority status for Protestants and Catholics in Northern Ireland, analysis was completed for the whole sample and separately for these two groups to examine potential differences in relations over time. 

## 2. Materials and Methods

Data were drawn from a six-wave longitudinal study on the effects of political violence on youth in Belfast. The last two waves, collected in 2011 and 2012, were utilized in the current study. In the current analyses, participants included 583 youth, aged 16.51 (*SD* = 2.01) and 17.23 (*SD* = 2.05) years old in each wave, roughly evenly split by gender (51% girls). Reflecting the demographics of Northern Ireland, all participants were White; there was a slight over-representation of the Protestant community (62%) compared to the Catholic community (38%). Seventy-eight percent of the sample was retained between waves. 

Families with a child between 10 and 17 years old were recruited to the larger study based on the neighborhoods in which they lived. To recruit participants, an expert demographer identified neighborhoods using data on historical politically motivated death rates during the Troubles, as well as sectarian crime reports to the Police Service of Northern Ireland [22]. All study areas were relatively homogeneous by Catholic/Protestant background, and they ranked in the lowest quarter of social deprivation (e.g., access to basic services, schools, education, and housing). Community leaders and families were sent letters informing them of the study and local interviewers from a market research firm followed up through door-to-door visits to assess eligibility and interest. All data were collected by face-to-face interview in the participants’ homes with youth interviews lasting approximately 45 min. The family received modest compensation at each time point. Procedures were approved by the Human Subjects Review Boards at all participating universities. 

The Exposure to Sectarian Antisocial Behavior scale (SAB) is a 12-item scale that assesses intergroup tension and violence and includes politically motivated events such as name calling by people from the other community, stones or objects thrown over walls, and deaths or serious injury caused by the other community. Participants indicated the frequency of SAB using a 5-point Likert scale from 0 = “*not in the last 3 months”* to 4 = “*every day*”. Higher scores represent more experiences with sectarian antisocial behavior in the three months prior to data collection. The SAB was developed locally through focus groups and a two-wave pilot test in Northern Ireland [23]. The internal consistencies of the waves used in the current study were good (0.91 and 0.94).

To assess participants’ exposure to non-politically motivated tension and violence participants completed the 7-item nonsectarian antisocial behavior scale (NAB). The same procedure that was used to develop the SAB was used to develop the NAB [23]. Example items of the NAB include having awareness of home break-ins, robberies, or murders in the community occurring in the 3 months prior. Cronbach’s alphas for the waves used in the current study were acceptable (0.79 and 0.73). 

To measure mental health problems, participants completed the 12-item version of the General Health Questionnaire (GHQ). The GHQ was developed as a screening instrument to identify psychological morbidity in primary care settings [24]. The GHQ is sensitive to psychological distress as it is commonly used as a screening instrument for depression and non-psychiatric morbidity [25]. Participants respond to statements about how they have been feeling recently (e.g., Have you recently lost much sleep over worry). Response options include a 4-point Likert scale ranging from much less than usual to much more than usual. The 12-item GHQ has been shown to be both reliable and valid amongst community samples in Northern Ireland [26,27]. Internal consistenciws for the two waves of data used in the present study were 0.90 and 0.92.

### Statistical Analysis

To model regression intercepts and slope coefficients as a smooth function of time, all analyses were modeled using the TVEM SAS Macro [28]. TVEM uses a spline-based approach to split a function of time into intervals that are based on several dividing points (knots). The major advantage of this approach is the user does not have to specify the shape of change and instead each interval is estimated using polynomial terms. Given this approach to estimating change, complex patterns that cannot be captured with simple linear or quadratic forms can emerge from the data. 

The results provided by the analysis are primarily graphical in nature with curvilinear estimates of the intercepts and slope functions. In the current analysis, change in the main outcome (GHQ scores) over continuous ages, along with the values of the coefficients representing associations between the main predictors of interest (SAB and NAB) at each age are presented graphically. In other words, the figures for coefficients for SAB and NAB represent how the relation between these variables and GHQ change through adolescence. Confidence bands (95%) are also included. If the confidence interval does not include zero at a particular point in time, a significant relation is indicated; if the confidence intervals do not overlap over time, a significant slope is indicated [29]. Gender and ethno-political group background were included as time in-variant control variables. For all models the P-spline (penalized truncated power spline) method was presented as P-spline utilizes information criteria to balance model fit with parsimony, thus, automatically selecting the number of knots to create the best model that does not overfit the data.

## 3. Results

### 3.1. Descriptive Statistics

Means, standard deviations, and possible ranges are presented in Table 1 by age by creating an age grouping variable. Just for purposes of presenting the descriptive statistics in this way, participants were binned such that anyone aged 10.50–11.49 was put in the 11-year-old group, 11.5- to 12.49 year olds were put in the 12-year-old group, and so on. It should be noted that when different binning ranges were used (e.g., ages 11–11.99 were put in the 11 group, 12–12.99 were put in the 12 group, etc.), the values for the means and standard deviations did change in some age groups. This further demonstrates the value of scaling age to the month and looking at age more continuously.

### 3.2. Time Varying Effects for the Full Sample

**Figure 1:**
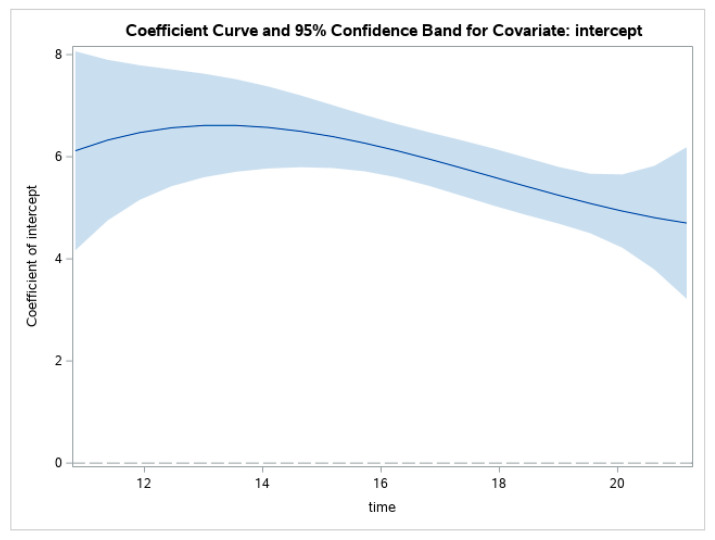
Change in GHQ scores through adolescence for the whole sample.

Figure 1 represents the changing value of the outcome variable, GHQ scores, which appears to be significant (i.e., the confidence interval does not include zero) and relatively stable from age 12 to about age 14, at which point it starts to decrease. The large confidence bands at the beginning and end of the covered age period are expected given the fewer observations in the data at those ages. It should be noted that given the overlapping confidence intervals throughout the figure, a change in value of the intercept is not significant.

**Figure 2:**
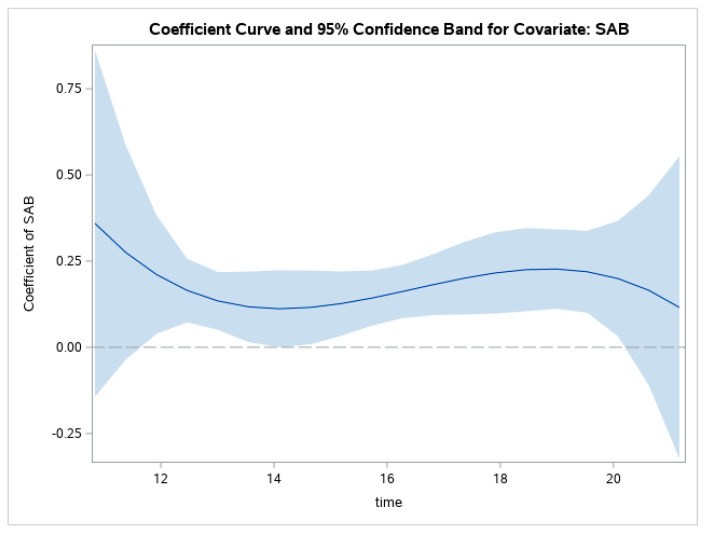
Changes in relations between SAB and GHQ through adolescence.

The line in Figure 2 represents the value of the regression coefficient between SAB and GHQ. Figure 2 suggests the relationship between SAB and GHQ becomes positive and stronger between ages 13 and 14, increasing until around age 19, at which point it decreases.

**Figure 3:**
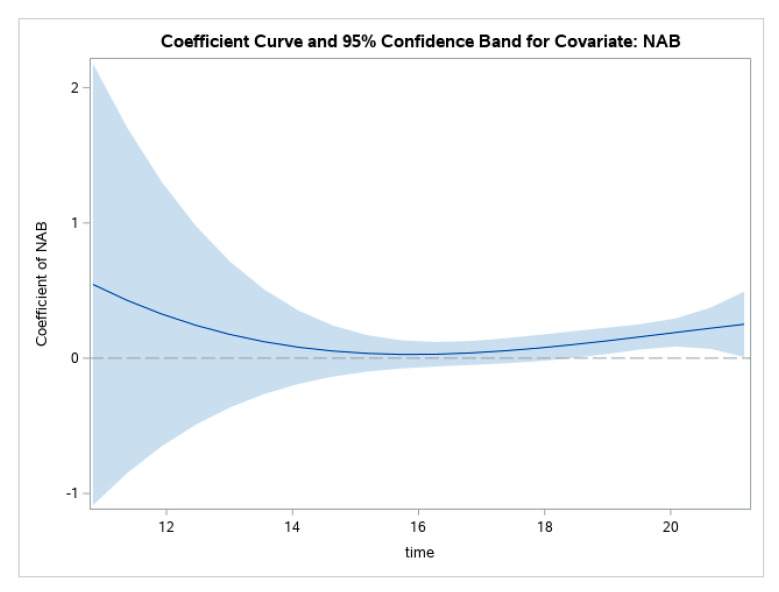
Changes in relations between NAB and GHQ through adolescence.

Similarly, the line in Figure 3 represents the value of the coefficient between NAB and GHQ over continuous age. This figure shows that, for the whole sample, there is a non-significant relation between NAB and GHQ throughout adolescence until around age 19.

### 3.3. Time Varying Effects for Catholics and Protestants Separately

**Figure 4:**
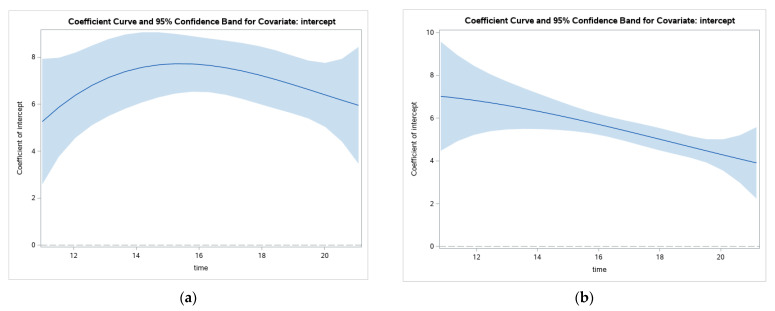
Changes in GHQ scores through adolescence for Catholics (**a**) and Protestants (**b**).

We also examined changes in the values of the coefficients over age for Catholics and Protestants separately. Looking at changes in GHQ scores (Figure 4), the figure suggests decreases over the age periods for both groups but different shapes of change. The line representing change in Catholic participants’ GHQ scores suggests an increase in mental health problems until around age 14, at which point, they start to decrease slightly. The figure for Protestants suggests a more linear downward slope of decreasing GHQ scores from before 12 through adolescence. Again, given the wide confidence band, the significance of this overall pattern must be interpreted with caution and again, could suggest variability between groups that is not accounted for.

**Figure 5:**
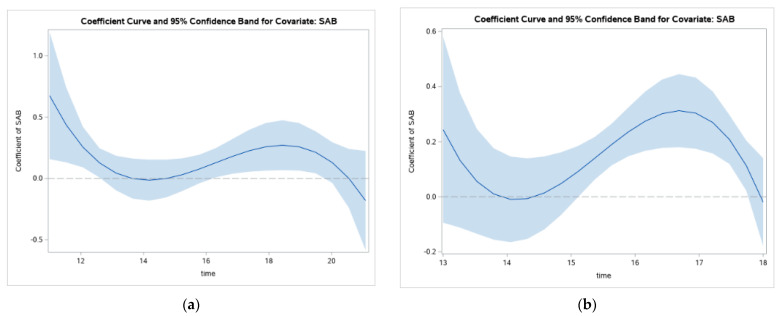
Changes in relations between SAB and GHQ scores through adolescence for Catholics (**a**) and Protestants (**b**).

Examining how the association between SAB and GHQ changes with age suggests a similar shape of change for both groups (Figure 5), but differences in timing and width of the confidence intervals. For both groups, there appears to be a decreasing relation between SAB and GHQ from early adolescence to age 14, at which point it starts to increase again until age 17 or 18. The exact ages modeled here differ slightly for the two groups. For Catholics, the full age range was used. For Protestants, the output utilizing the whole sample resulted in a figure with an extremely wide tail in the early ages that impacted the scaling of the Y axis. The figure shown here restricted the sample to 13–18 year olds. From this output, you can see the period between 13 and 14 for Catholics and 13 and 15 for Protestants, the line representing the coefficient is becoming stronger and significant until it appears to decrease again around age 19 for Catholics and 17 for Protestants.

**Figure 6:**
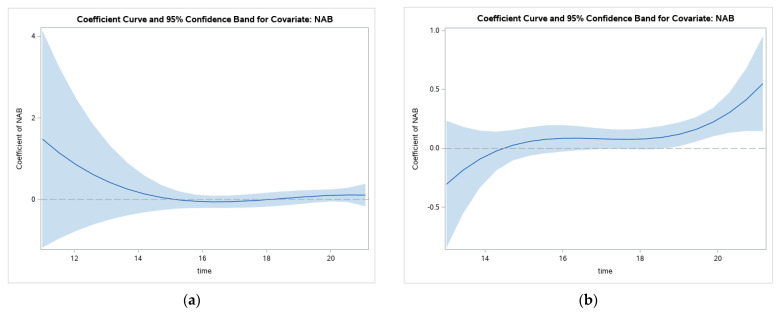
Changes in relations between NAB and GHQ scores through adolescence for Catholics (**a**) and Protestants (**b**).

Similar to the findings for the overall sample, looking at the curves for the coefficient for NAB and GHQ symptoms for Catholics and Protestants separately suggests that throughout most of adolescence, NAB does not predict GHQ scores (Figure 6). The tail end of the curve for Protestants suggests a significant relation starting around 19. Again, the initial output for Protestants made this emergence of the significant relation difficult to see so the figure below includes those 13 and older to adjust the y axis, so that the change at the end of adolescence can be seen more clearly.

## 4. Discussion

The goal of the current study was to utilize an exploratory method of data visualization to examine age-related changes in the relation between exposure to political tension and violence and mental health problems in adolescents from Belfast, Northern Ireland. The results suggest that mental health problems, as measured by the GHQ, appear fairly stable throughout this age period, with some indication that increases in problems are occurring in the preadolescent period, then showing some signs of decreasing after age 14. These findings are consistent with previous studies suggesting that internalizing problems, such as depression and anxiety, begin to increase during early adolescence and then start to decrease into adulthood [6]. The confidence bands at the beginning and end of the age period collected here are quite wide as is often the case with these models given the limited data being used to estimate the curve at these points. Given the width of the confidence intervals, the increases and decreases in the trend lines should be interpreted with caution and importantly may suggest variability between groups that may help to capture more precise estimates of change. 

Looking at changes in relations between exposure to continued sectarian tensions in Belfast suggests an increased strength of association with mental health problems from between the ages of 14 until age 17–18, at which point the strength of the relationship decreases. One potential explanation for this increase in relations is the increase in identity salience of peer relationships that become more important through adolescence [30,31]. Given the group-based nature of ethno-politically motivated tension and violence in Belfast, along with the history of the meaning of that violence for group members, it follows that increasing group-based identity salience could strengthen relations between exposure to this form of conflict and mental health problems. A second explanation could be that it is the accumulation of exposure over time that increases the risk for mental health problems. In other words, the accumulation of exposure to political tension and violence along with related stressors may accumulate over years to impact mental health problems, as coping strategies break down and systems become overwhelmed [32].

The results for non-sectarian antisocial behavior show non-significant associations with mental health problems until the end of adolescence. Breaking this down by Catholic and Protestant groups suggests the emergence of significant associations is only present with Protestants and not Catholics. It is important to note that this is not a direct test of differences between the groups so the different patterns should be interpreted with caution. Overall, this pattern suggests that sectarian antisocial behaviors have stronger associations with mental health problems compared with exposure to ordinary crime and antisocial behavior that is not politically motivated. Few studies have examined the impacts of these different forms of tension and violence and, more commonly, assessed exposure to violence without differentiating between political-motivated and ordinary crime (e.g., [33]; see [34,35] for exceptions); thus, these findings represent evidence that separating the impacts of these types of violence may have utility. Moreover, the evidence for potential differences in the impacts of exposure to non-politically motivated tension and violence may signal different impacts for Protestants and Catholics. One explanation for this difference could be related to differences in education between the two groups. For example, gaps in undergraduate and post-graduate enrollments persisted with Protestants, particularly Protestant males, seeking these degrees less often than Catholics [36]. These patterns could signal additional challenges facing Protestants during this transition out of compulsory education. 

As always, the contributions of this research must be considered with clear acknowledgement of the limitations of the study and analysis. All data were self-reported; thus, biases in memory of events experienced may correlate with existing mental health problems or other self-report biases may have impacted the findings. Further, the nature of the analysis limits claims of cause or directionality. Although the analysis was selected with intention as a complement to existing methods, it should also be acknowledged that this approach to data analysis is exploratory in nature—the patterns of change emerge from the data without placing restrictions on what that change looks like. While this is an advantage regarding the ability to see semi-parametric forms of change emerge, as with many forms of exploratory data analysis, there is a risk of modeling noise in the data that would limit replicability in other samples. 

## 5. Conclusions

Identifying dynamic patterns of change in relations between predictors and outcomes is important for prevention and intervention efforts that are at the heart of the developmental psychopathology perspective [1,2]. Using exploratory approaches to age-based changes allows for the emergence of patterns in the data that might indicate key timing for prevention or intervention. Moreover, examining the contextual and individual processes that may explain these dynamic patterns can help clarify the content of such programs. The findings from the current study suggest that the association between sectarian or politically motivated tension and violence have increasing association to mental health problems for middle adolescence in Belfast. This set of findings, if replicated, might suggest targeting intervention programs for youth in these communities prior to age 13 or 14. These findings are also consistent with the emerging importance of social relationships through early adolescence and may suggest programming that specifically focuses on exposure to identity-related conflict as it relates to internalizing problems. School-based anti-bullying programs, for example, might include identity-related themes. Additional new questions might test the idea that it is the accumulation of exposure in the years leading up to this period that is causing the increased association with mental health problems in mid-adolescence, along with the identity-related changes that are occurring through the adolescent period. Looking at the results for exposure to non-politically motivated violence suggests later associations with mental health and group-based processes that should be considered and may point to other contexts (e.g., education and employment) that may be relevant to these processes. For example, the current results suggest a link between exposure to non-sectarian forms of conflict in emerging adulthood for Protestants in particular. Given the transition out of compulsory education during this developmental stage, community programming, as opposed to school-based programming, focusing on the connection between conflict and mental health, may be beneficial at this stage. While the results of the current study cannot directly pinpoint why a certain association might exist at a given age, it can help identify new questions to be answered.

## Figures and Tables

**Table 1:** Means, standard deviations and possible ranges for main study variables.

Age	SAB	NAB	GHQ
11	0.38 (1.06)	0.86 (1.21)	6.00 (0)
12	3.44 (9.96)	0.71 (1.25)	6.22 (2.77)
13	0.97 (3.13)	2.51 (2.45)	7.43 (3.78)
14	1.52 (4.07)	2.56 (3.29)	7.16 (4.20)
15	2.18 (5.60)	3.29 (3.47)	6.30 (2.98)
16	3.48 (6.91)	4.41 (4.25)	6.65 (3.81)
17	2.69 (6.38)	4.68 (4.47)	7.14 (4.44)
18	1.66 (4.82)	5.30 (4.66)	5.62 (2.91)
19	1.58 (4.82)	5.78 (4.27)	5.50 (3.26)
20	1.51(3.90)	5.55 (4.19)	6.68 (5.07)
21	1.19 (2.68)	6.25 (4.64)	5.75(2.70)
Possible Range	0–36	0–28	0–48

## Data Availability

The data and related codes that support the findings of this study are available on request from the corresponding author.
